# The Investigation of Hybrid PEDOT:PSS/β-Ga_2_O_3_ Deep Ultraviolet Schottky Barrier Photodetectors

**DOI:** 10.1186/s11671-020-03397-8

**Published:** 2020-08-14

**Authors:** Tao Zhang, Yixian Shen, Qian Feng, Xusheng Tian, Yuncong Cai, Zhuangzhuang Hu, Guangshuo Yan, Zhaoqing Feng, Yachao Zhang, Jing Ning, Yongkuan Xu, Xiaozheng Lian, Xiaojuan Sun, Chunfu Zhang, Hong Zhou, Jincheng Zhang, Yue Hao

**Affiliations:** 1 State Key Discipline Laboratory of Wide Band Gap Semiconductor Technology, School of Microelectronics, Xidian University, Xi’an, 710071 China; 2 Shaanxi Joint Key Laboratory of Graphene, School of Microelectronics, Xidian University, Xi’an, 710071 China; 3 China Electronics Technology Group Corporation No. 46 Research Institute, Tianjin, 300220 China; 4 State Key Laboratory of Luminescence and Applications, Changchun Institute of Opeics, Chinese Academy of Sciences, Changchun, 130033 China

**Keywords:** β-Ga_2_O_3_, PEDOT:PSS, Hybrid Schottky diodes, Photodetector

## Abstract

In this paper, the hybrid β-Ga_2_O_3_ Schottky diodes were fabricated with PEDOT:PSS as the anode. The electrical characteristics were investigated when the temperature changes from 298 K to 423 K. The barrier height *ϕ*_*b*_ increases, and the ideality factor *n* decreases as the temperature increases, indicating the presence of barrier height inhomogeneity between the polymer and β-Ga_2_O_3_ interface. The mean barrier height and the standard deviation are 1.57 eV and 0.212 eV, respectively, after taking the Gaussian barrier height distribution model into account. Moreover, a relatively fast response speed of less than 320 ms, high reponsivity of 0.6 A/W, and rejection ratio of *R*_254 nm_/*R*_400 nm_ up to 1.26 × 10^3^ are obtained, suggesting that the hybrid PEDOT:PSS/β-Ga_2_O_3_ Schottky barrier diodes can be used as deep ultraviolet (DUV) optical switches or photodetectors.

## Introduction

Many research groups have paid lots of attention to a new ultrawide bandgap semiconductor of β-Ga_2_O_3_ as a potential material for deep ultraviolet (DUV) photodetectors [1–7], high voltage, and high power devices for its wide band gap (4.8–4.9 eV), high breakdown electric field (8 MV/cm), and chemical stability [8–11]. In addition, it is simple to cleave β-Ga_2_O_3_ into nano-membranes or thin belts [12, 13] for its unique property of the large lattice constant along [100] direction .Various metals, such as Cu [14], Pd [15], Pt [11, 16–19], Au [15, 20], Ni [16, 21–23], and TiN [18], were used to investigate the electrical characteristics of β-Ga_2_O_3_ Schottky barrier diodes (SBD). However, the Schottky diodes fabricated with some polymer and the electrical characteristics have not been reported yet. Among all the organic materials, PEDOT:PSS is one of the transparent hole-conducting polymers, whose conductivity is up to 500 S/cm and work function is up to 5.0 ~ 5.3 eV, close to Au and Ni [23–25]. Furthermore, the PEDOT:PSS film can be formed only by spin-coating onto the substrate and subsequent baking in air. There are some investigations in regard to the transparent Schottky contact of PEDOT:PSS on ZnO single crystalline substrate and GaN epilayer, exhibiting rectifying good properties and photoelectrical or photovoltaic characteristics [26–29].

In this work, the hybrid Schottky diode was fabricated with PEDOT:PSS polymer and the mechanically exfoliated β-Ga_2_O_3_ flakes from the high quality β-Ga_2_O_3_ substrate. The electrical characteristics of the diodes were investigated in the temperature region between 298 K and 423 K. Furthermore, the I–V measurements under the UV illumination were carried out, the responsivity was measured, and the transient behavior of the photocurrent was also analyzed.

## Experimental Methods

**Fig. 1:**
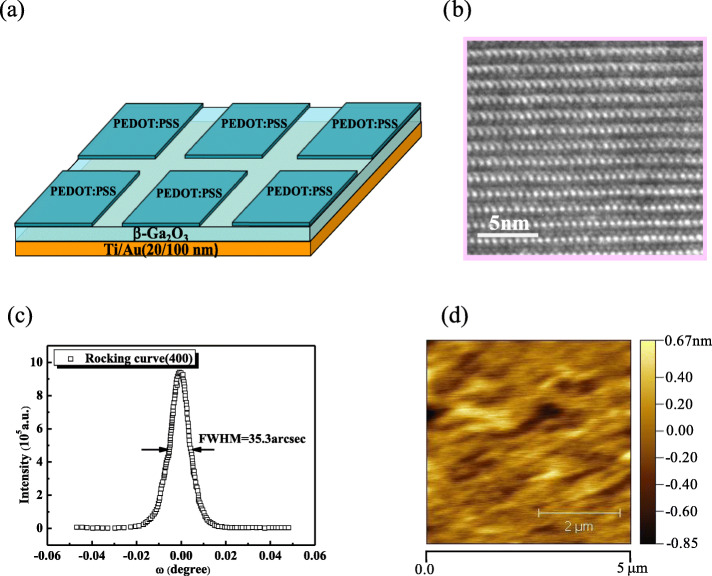
Schematic diagram of the hybrid PEDOT:PSS/β-Ga_2_O_3_ Schottky diode (**a**), HRTEM image (**b**), HRXRD rocking curve of the (400) plane (**c**), AFM image of β-Ga_2_O_3_ flake obtained from β-Ga_2_O_3_ substrate by mechanically exfoliation, showing a high crystal quality and smooth surface (**d**)

The β-Ga_2_O_3_ flakes with the thicknesses of 15–25 μm were mechanically exfoliated from the (100) β-Ga_2_O_3_ substrate with the electron concentration of 7 × 10^16^ cm^−3^. For the electron density is 2–3 orders of magnitude higher than that in the unintentionally doped Ga_2_O_3_ epilayer deposited on sapphire substrate in [30] and the highly conductive PEDOT:PSS films was used in this paper, so the pn heterojunction was formed in [30] while Schottky junction was formed in this paper [30]. Figure 1a shows the schematic diagram of the hybrid PEDOT:PSS/β-Ga_2_O_3_ Schottky diode. The β-Ga_2_O_3_ flakes were cleaned in acetone, ethanol, and deionized water with ultrasonic agitation and then immersed into the HF: H_2_O (1:10) solution to remove surface oxides. Then, the deposition of Ti/Au(20 nm/100 nm) metal stack was carried out on the whole back side, and the rapid thermal processing at 470 °C under N_2_ atmosphere was conducted for 60 s to decrease the ohmic contact resistance. After spin coated onto the surface of β-Ga_2_O_3_ flake for three times, PEDOT:PSS was baked on an electric hotplate at 150 °C, and the baking duration was 15 min. Subsequently, isolated devices with the area of 1 mm × 2 mm were obtained. From the HRTEM image of Fig. 1b, we can observe that the atoms are regularly arranged and few atomic column misalignments are present, indicating a high crystal quality of the β-Ga_2_O_3_ flake. As shown in Fig. 1c, d, the FWHM of HRXRD is about 35.3 arcsec, and the root mean square (RMS) is estimated to be 0.19 nm, illustrating the superior crystal quality and smooth surface.

## Result and Discussion

### I–V Characteristics and Barrier Height

**Fig. 2:**
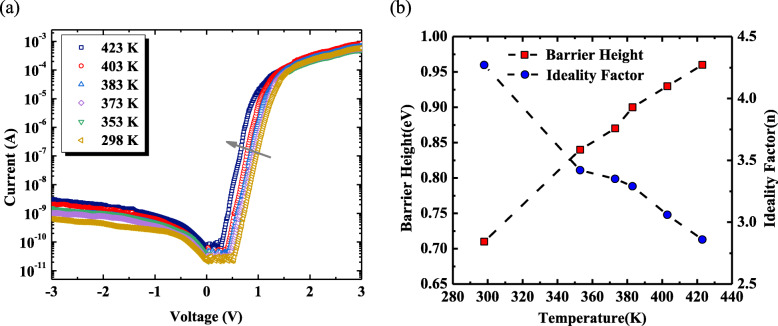
Temperature-dependent I–V characteristics of PEDOT:PSS/β-Ga_2_O_3_ SBDs from 298 to 423 K (**a**) and Schottky barrier height *ϕ*_b_ and ideality factor *n* of hybrid β-Ga_2_O_3_ SBD (**b**)

As presented in Fig. 2a, the I–V characteristics of the hybrid PEDOT:PSS/β-Ga_2_O_3_ Schottky barrier diodes were investigated when the temperature changes from 298 K to 423 K. The current increases monotonously with the temperature and the semi-log I–V curves show the linear behavior as the forward voltage bias less than 1.5 V. As the forward bias voltage further increases, the slope of the semi-log I–V curves gradually reduces, and the forward current approaches 6 ~ 8 × 10^−4^ A, indicating that the series resistance causes the I–V curve deviating from the linearity. In addition, the reverse leakage current is less than 10^−9^ A at – 3 V, and the *I*_on_/*I*_off_ ratio is up to 10^6^ at room temperature, illustrating a rectifying behavior as good as inorganic β-Ga_2_O_3_ Schottky diodes [11–15].

According to the equation $I={I}_s\left\{\exp \left[\frac{q\left(V-{IR}_s\right)}{nkT}\right]-1\right\}$ where *V* is the bias voltage, *T* and *k* are the absolute temperature and the Boltzmann constant, respectively. The ideality factor *n* and the reverse saturation current *I*_*s*_ can be extracted from the *y*-axis intercepts and the slopes of the linear extrapolation of the semi-log I–V curves at different temperatures. Although the ideality factor *n* of the ideal Schottky diode is equal to 1, it is always larger than 1 to some extent in actual device. The deviation of the thermal emission (TE) model becomes much greater as *n* increases. According to the expression ${\phi}_b=\frac{kT}{q}\ln \left[\frac{AA^{\ast }{T}^2}{I_s}\right]$, we can obtain the Schottky barrier height *ϕ*_*b*_ at different temperatures, as shown in Fig. 2b. The increase in temperature causes *ϕ*_*b*_ to increase from 0.71 eV to 0.84, 0.87, 0.90, 0.93, and 0.96 eV while *n* to decrease from 4.27 to 3.42, 3.35, 3.29, 3.06, and 2.86. For *n* much larger than 1, suggesting other conducting mechanisms, such as field effect or thermal field effect, contributing to the current transport and resulting in the difference between pure TE model and the I–V characteristics, which has been illustrated in the wide bandgap SBDs, including GaN and SiC [31–34].

**Fig. 3:**
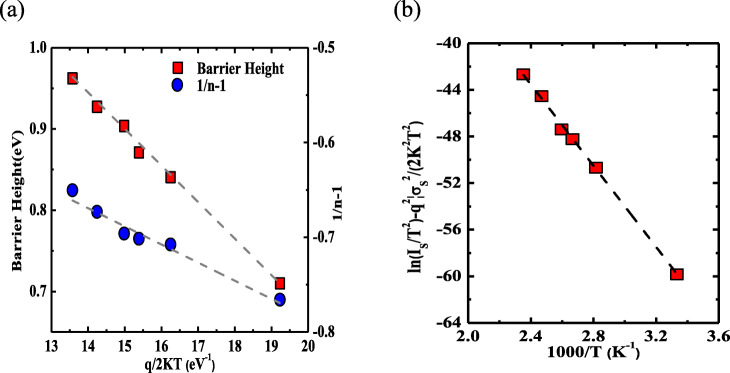
The variation of the SBH *ϕ*_b_ and (*n*^−1^ − 1) with *q*/2*KT* curves, $\overline{\phi_{b0}}$ and *σ*_*s*_ can be obtained (**a**), modified $\ln \left({I}_{\mathrm{s}}/{T}^2\right)-\left({q}^2{\sigma}_{\mathrm{s}}^2/2{k}^2{T}^2\right)$ versus 1000/*T* plot (**b**)

For *ϕ*_*b*_ and *n* are temperature-dependent, the inhomogeneity of barrier height should be considered at PEDOT:PSS and β-Ga_2_O_3_ interface. Considering the Gaussian distribution of the barrier height, the inhomogeneous barrier height may be described as ${\phi}_b=\overline{\phi_{b0}}\left(T=0\right)-\frac{q{\sigma}_s^2}{2 kT}$ and the variation of *n* with *T* is given by $\left(\frac{1}{n}-1\right)={\rho}_2-\frac{q{\rho}_3}{2 kT}$, where $\overline{\phi_{b0}}$ and *σ*_*s*_ are the mean barrier height and the standard deviation, respectively, *ρ*_2_ and *ρ*_3_ are the temperature-dependent voltage coefficients, and the voltage deformation of the Schottky barrier height (SBH) distribution was quantified by them (Fig. 3a). $\overline{\phi_{b0}}$ and *σ*_*s*_ can be calculated from the intercept and the slope of the *ϕ*_*b*_ versus *q*/2*kT* curve, about 1.57 eV and 0.212 eV, respectively. At the same time, *ρ*_2_ and *ρ*_3_ are evaluated to be 0.4 eV and 0.02 eV from the intercept and slope of the (1/*n* − 1) versus *q*/2*kT* plot. Compared with $\overline{\phi_{b0}}$, *σ*_*s*_ is not small, illustrating the existence of barrier inhomogeneity at PEDOT:PSS/β-Ga_2_O_3_ interface [35].

By considering the barrier height inhomogeneity, the relationship between the reverse saturation current *I*_*s*_ and the mean barrier height $\overline{\phi_{b0}}$can be modified as $\mathrm{In}\left(\frac{I_s}{T^2}\right)-\left(\frac{q^2{\sigma_s}^2}{2{k}^2{T}^2}\right)=\mathrm{In}\left({AA}^{\ast}\right)-\frac{q\overline{\phi_{b0}}}{kT}$. It can be discerned from Fig. 3b that the plot of the $\ln \left({I}_{\mathrm{s}}/{T}^2\right)-\left({q}^2{\sigma}_{\mathrm{s}}^2/2{k}^2{T}^2\right)$ versus 1/*kT* is a straight line, from which we can extract the effective Richardson constant A^*^ of 3.8 A cm^−2^K^−2^, one order magnitude smaller than the theoretical Richardson constant of 40.8 A cm^−2^K^−2^ with the β-Ga_2_O_3_ effective mass of m* = 0.34 m_0_ [36, 37]. Thus, the temperature-dependent *ϕ*_*b*_ and *n*, in other words, the Gaussian distribution of the barriers over SBHs can be used to explain the barrier inhomogeneity at the PEDOT:PSS/β-Ga_2_O_3_ interface.

### Characteristics of UV Photodetector

**Fig. 4:**
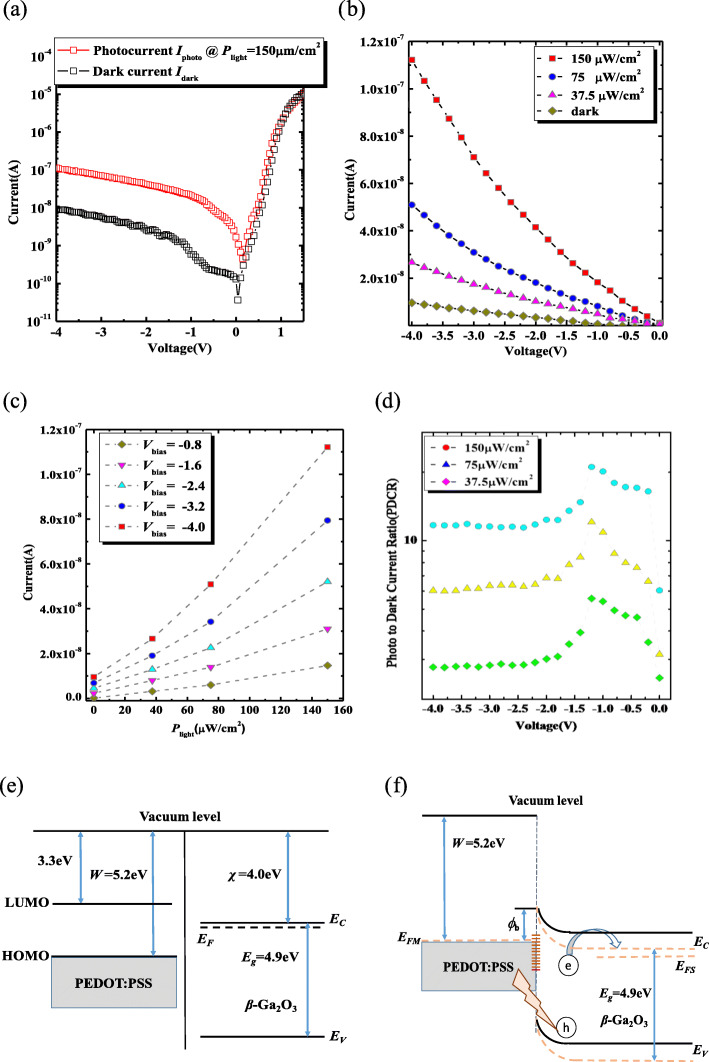
Relationship between Photocurrent *I*_photo_@150 μW/cm^2^, dark current *I*_dark_, and bias voltage *V*_bias_ (**a**), plots of *I*_photo_ versus *V*_bias_ under different *P*_light_ (**b**), linear *I*_photo_ as a function of *P*_light_ (**c**), curves of photo to dark current ratio (PDCR) under different *P*_light_ (**d**), band diagram of PEDOT:PSS and β-Ga_2_O_3_ before contact (**e**), band diagram of PEDOT:PSS and β-Ga_2_O_3_ under the reverse bias after contact, the condition without applied voltage and the condition with the reverse bias are shown by the solid line and the dashed line, respectively (**f**)

As described above, the hybrid β-Ga_2_O_3_ Schottky diode exhibits a good rectifying characteristics; the ratio of *I*_on_/*I*_off_ up to 10^6^ in dark state at room temperature. The lower dark current *I*_dark_ of 9.4 nA@*V*_bias_ = − 4 V can be determined from Fig. 4a, indicating a lower noise characteristic. While under the normal incidence of 254 nm wavelength with the photodensity of 150 μW/cm^2^, the photocurrent *I*_photo_ reaches 112 nA@*V*_bias_ = − 4 V. In addition, the photodetector shows a weak photovoltaic effect with a photocurrent of 0.45 nA at 0 V and an open-circuit voltage (*V*_oc_) of 0.15 V, much less than 0.9 V in reference [38], which may be attributed to the carrier density difference and the resulting Fermi level variation. Figure 4b represents the linear *I*_photo_ versus *V*_bias_ at various *P*_light_. The device shows the dependence of *I*_photo_ on the *P*_light_, and the *I*_photo_ increases non-linearly with the *P*_light_, in other words, at different *V*_bias,_ the plots of *I*_photo_ versus *P*_light_ demonstrate an obvious superlinear behavior, as shown in Fig. 4c. In order to elucidate the mechanism of the superlinear behavior, Fig. 4e presents the energy diagram of the PEDOT:PSS and β-Ga_2_O_3_ before contact. The electron affinity and the bandgap of β-Ga_2_O_3_ are 4.0 eV and 4.9 eV, respectively. The lowest unoccupied molecular orbital (LUMO) is 3.3 eV, and the highest occupied molecular orbital of PEDOT:PSS is 5.2 eV [39]. As they came to contact, a Schottky barrier was formed. When the device is illuminated and the reverse bias is applied to the electrodes of the Schottky diodes, the photo generated electron-hole pairs are separated rapidly by the electric field and the holes drift to the anode while the electrons to the cathode, as shown in Fig. 4f. For the presence of traps at the PEDOT: PSS/β-Ga_2_O_3_ interface, the holes are trapped at the interface states and produce net positive charges, reducing the effective Schottky barrier height, more carriers flowing across the Schottky junction, and improving the *I*_photo_. Figure 4d presents the photo to dark current ratio (PDCR) curves under different *P*_light_. As the voltage bias shifts from

0V to − 1.2V, the PDCR increases gradually and then decreases with the voltage bias becoming more negative, the higher PDCR above 20 is achieved at a *V*_bias_ of − 1.2 V and a *P*_light_ of 150 μW/cm^2^.

**Fig. 5:**
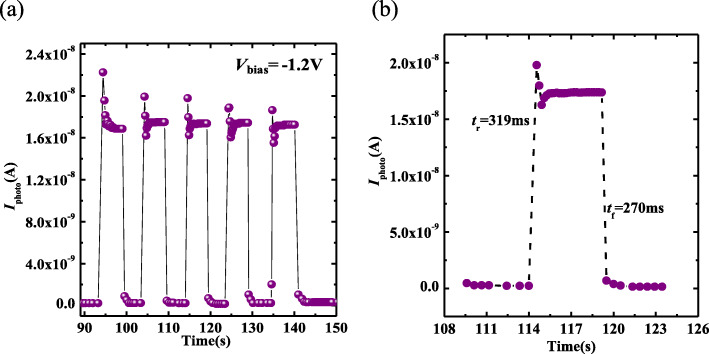
Multi-cycles (**a**) and single cycle (**b**) of time-dependent *I*_photo_ of the hybrid PEDOT:PSS/β-Ga_2_O_3_ Schottky barrier photodetector at the *V*_bias_ = − 1.2 V, the rise time and decay time are determined to be 319 ms and 270 ms, respectively

The time-dependent photoresponse characteristics of hybrid photodetector are studied by using square wave light with a period of 10 s under the *V*_bias_ of − 1.2 V and a *P*_light_ of 150 μW/cm^2^. After several illumination cycles, devices reach the stable on-state *I*_photo_ at the given *P*_light_ and *V*_bias_, as represented in Fig. 5a. The rise time and decay time are 319 ms and 270 ms [40, 41], respectively, much less than those of devices fabricated on epitaxial β-Ga_2_O_3_ films or β-Ga_2_O_3_ flakes [35, 42, 43] but longer than the data in [31]. For the existence of double heterojunction in [31], PEDOTT:PSS/Ga_2_O_3_ upper junction and Ga_2_O_3_/p-Si lower junction, the photogenerated carriers can be separated more effectively by the double built-in electric fields than the only one PEDOTT:PSS/Ga_2_O_3_ junction in this paper. Therefore, less carriers can be captured by the defects in [31], resulting in the shorter rise time and decay time. Furthermore, the overshooting feature can be observed from the shapes of photoresponse curves with a wedgy head at the lower *P*_light_ of 150 μW/cm^2^ than that occurred at the *P*_light_ of 600 μW/cm^2^ in [30] for the effective collection of photogenerated carriers under the reverse bias of − 1.2 V rather than 0 V.

**Fig. 6:**
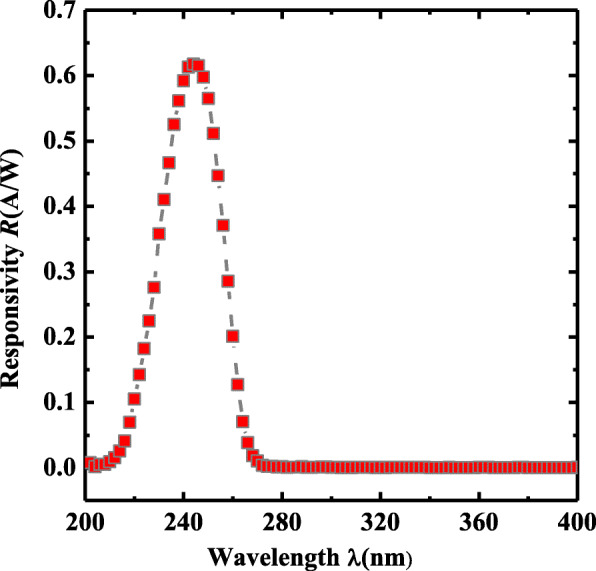
Responsivity versus wavelengths for the PEDOT:PSS/Ga_2_O_3_ hybrid photodetectors at *V*_bias_ =-1.2 V

Figure 6 depicts the responsivity characteristics versus the illumination optical *λ* under the *V*_bias_ of − 1.2 V. The maximum responsivity *R*_max_ of 0.62 A/W is achieved at a *λ* of 244 nm and the corresponding external quantum efficiency(EQE) of 3.16 × 10^2^% calculated by the expression EQE = *hcR*_max_/(*eλ*), much higher than that obtained in [30, 38] for the effective collection of photogenerated carriers, where *R*_max_ is the peak responsivity, and *h* is the Plank constant. *e* and *λ* are the electronic charge and the illumination wavelength, respectively. As the wavelength is longer than 290 nm, the photoresponsivity is lower than 1 × 10^−3^, illustrating a much better spectral selectivity in the hybrid β-Ga_2_O_3_ devices. At the same time, the rejection ratio of *R*_254 nm_/*R*_400 nm_ is determined to be 1.26 × 10^3^. Compared with the reported inorganic Ga_2_O_3_ photodetector [43–49], the hybrid device possesses a higher photoresponsivity, faster response speed and larger UV/visible rejection ratio, implying a promising solar blind photodetectors with high performance.

## Conclusions

We have fabricated PEDOT:PSS/β-Ga_2_O_3_ hybrid Schottky barrier diode. The Schottky barrier height *ϕ*_*b*_ and ideality factor *n* are dependent on temperature, indicating that the Schottky barrier height was inhomogeneous at PEDOT:PSS/β-Ga_2_O_3_ interface. The mean barrier height and standard deviation can be evaluated to be 1.57 eV and 0.212 eV, respectively, based on the Gaussian barrier height distribution model. Furthermore, the characteristics of PEDOT:PSS/β-Ga_2_O_3_ DUV Schottky barrier photodetectors were also investigated. A higher responsivity of 0.6 A/W, rejection ratio of *R*_254 nm_/*R*_400 nm_ = 1.26 × 10^3^, EQE of 3.16 × 10^4^% and a faster response speed of less than 320 ms are achieved, suggesting that the hybrid Schottky barrier diodes can be used as DUV optical switches or photodetectors.

## Data Availability

All data is available from the authors via a reasonable request.

## Abbreviations

AFM: Atomic force microscope
DUV: Deep ultraviolet
EQE: External quantum efficiency
FWHM: Full-width half maximum
HRTEM: High-resolution transmission electron microscopy
LUMO: Lowest unoccupied molecular orbital
PDCR: Photo to dark current ratio
RMS: Root mean square
SBDs: Schottky barrier diodes
TE: Thermal emission
